# Activity of granulomatosis with polyangiitis and its correlation with mTOR phosphoproteomics in neutrophils

**DOI:** 10.3389/fimmu.2023.1227369

**Published:** 2023-08-31

**Authors:** Marcin Surmiak, Katarzyna Wawrzycka-Adamczyk, Joanna Kosałka-Węgiel, Anna Włudarczyk, Marek Sanak, Jacek Musiał

**Affiliations:** ^1^ Department of Internal Medicine, Jagiellonian University Medical College, Krakow, Poland; ^2^ Department of Rheumatology and Immunology, University Hospital, Krakow, Poland; ^3^ Department of Intensive Care and Perioperative Medicine, Jagiellonian University Medical College, Krakow, Poland

**Keywords:** granulomatosis with polyangiitis, mammalian target of rapamycin, neutrophil extracellular traps, autophagy, neutrophils, mitochondrial DNA

## Abstract

**Introduction:**

Granulomatosis with polyangiitis (GPA) is a small vessel vasculitis with a complex pathomechanism. Organ damage in GPA is also mediated by extracellular trap formation (NETosis). We analyzed the functional status of phosphoproteins modulating NETosis in neutrophils by the mammalian target of rapamycin (mTOR) pathway in GPA along with NETosis biomarkers.

**Methods:**

Phosphoproteins levels measured in isolated neutrophils from 42 patients with GPA (exacerbation n=21; remission n=21) and 21 healthy controls were compared to serum biomarkers of the disease.

**Results:**

Neutrophils in active disease manifested lowered levels of phosphorylated mTOR^(Ser2448),^ PTEN^(Ser380)^ and ULK1^(Ser555)^, whereas phosphorylated GSK-3α/β^(Ser21/Ser9)^ was elevated. Exacerbation of GPA was characterized by elevated neutrophil dsDNA in serum, circulating mitochondrial DNA, and DNA-MPO complexes. A significant negative correlation between mTOR or PTEN phosphoproteins and biomarkers of GPA activity was also present, reflecting the clinical activity score of GPA. Positive correlations between phosphorylated GSK-3 α/β and circulating mtDNA, DNA-MPO complexes, neutrophil-released dsDNA, or circulating proteins were also significant. Increased serum levels of IGFBP-2, TFF-3, CD147, and CHI3L1 accompanied GPA exacerbation, whereas DPP-IV levels were the lowest in active GPA. Using a principal component analysis basigin, PTEN and mTOR had the highest loadings on the discrimination function, allowing classification between active, remission, and control subjects with 98% performance.

**Conclusions:**

We present evidence that inhibited mTOR signaling accompanies NETosis in patients with GPA. The functional status of phosphoproteins suggests simultaneous activation of NETosis and autophagy. These results give rise to the study of autophagy as a mechanism underlying granuloma formation in GPA.

## 1 Introduction

Granulomatosis with polyangiitis (GPA) is small vessel vasculitis with a complex pathomechanism and neutrophil as the key effector cell. Most patients with GPA have antineutrophil cytoplasmatic autoantibodies (ANCA) against proteinase 3 (PR3). Especially in a generalized GPA, PR3-ANCA are involved in tissue damage (1). Besides this humoral hallmark, the dysfunction of regulatory T-lymphocytes characterizes active GPA by higher levels of CD4+ and concurrent monocytic activation (2). The formation of neutrophil extracellular traps (NETosis) is an innate mechanism of pathogen elimination that is activated in some autoimmune diseases such as lupus or vasculitides (3). Among modulators of NET formation, the mammalian target of rapamycin (mTOR) is operating in two intracellular complexes (mTORC1 and mTORC2). It is a member of the phosphatidylinositol 3-kinase (PI3K)-like kinase family regulated by phosphorylation and acting on several intracellular processes, including the production of pro-inflammatory cytokines and autophagy (4). In the present study, we analyzed phosphoproteins involved in mTOR pathways of neutrophils from patients with GPA and correlated them with biomarkers of NETosis. We also analyzed serum levels of differentially expressed circulating proteins potentially participating in mTOR signaling, NETosis, and autophagy.

## 2 Methods

### 2.1 Patients and study design

In this non-randomized observational single-center study, we enrolled 42 patients with GPA (active stage n=21 or remission n=21) and a control group of 21 age- and sex-matched healthy volunteers (HC) (**Table 1**). GPA was diagnosed according to ACR 1990 (5) but including the 2012 Revised Chappel Hill Consensus Nomenclature (6). All GPA patients were positive for anti-PR3 IgG antibodies and negative for anti-MPO IgG antibodies. Disease activity was evaluated using the Birmingham Vasculitis Activity Score (BVAS, version 3) and organ damage was ascertained using the Vasculitis Damage Index (VDI). Remission was defined as lack of symptoms of active disease (BVAS = 0). Exacerbation of the disease was identified by the presence of new or reemerging signs and symptoms of vasculitis (confirmed by the BVAS > 6), requiring intensification of immunosuppressive therapy according to EULAR recommendations (7). Patients with cancer, kidney failure requiring dialysis, or infection were excluded from this study. Blood samples from all patients during the active stage of GPA were collected before the onset of intensive treatment with high-dose corticosteroids or immunosuppressants. In the group of patients with remission of GPA, 19 patients (90%) were on remission maintenance treatment, 19 patients (90%) received oral glucocorticosteroids (up to 8 mg/day), and 7 patients (33%) were on disease-modifying anti-rheumatic drugs (3 on methotrexate and 4 on azathioprine). Peripheral blood samples were collected without anticoagulant or with sodium citrate anticoagulant using a blood collection system (Venous Blood S-Monovette, Sarstedt, Germany) between October 2016 and August 2019. Serum and plasma were separated using standard laboratory procedures, aliquoted, and frozen at -80° C for further analyses. Laboratory tests (CBC, CRP, anti-PR3/anti-MPO IgG level) were performed on all study participants at the time of blood sampling. Written informed consent was obtained from all study participants and the study protocol was accepted by the ethics committee of Jagiellonian University.

**Table 1:** Selected characteristics of the study participants.

	Active GPA	Remission of GPA	Healthy control
N Newly diagnosed Exacerbation	21 11 10	21 0 0	21
Age (mean ± SD)	57.4 ± 12.9	56.2 ± 13.1	54.1 ± 12.7
Sex (F/M)	12/9	11/10	12/9
BVAS (min-max)	4-35	0	–
VDI (min-max)	2-8	5-10	–
Organs involved			–
Ear, nose, throat Lungs Kidneys Joints Skin/mucous membr. Nervous system	18 17 18 16 15 11	14 3 0 0 0 2	
GC treatment (YES/NO)	13/7	19/2	–
GC dose [mg/day] (min-max)	4-24	4-8	–
CYC cumulative dose^#^	0 - 37	5-40	–
cANCA [IU/mL] (min-max)	12-197	<2-123	–
CRP [mg/L]	5-270	<5-7	0
Procalcitonin [ng/mL]	<0.05	<0.05	<0.05
PBMC [10^3^/µL]	11.5 ± 3.6^*^	8.3 ± 2.6	6.5 ± 4.5
PMN [10^3^/µL]	8.2 ± 4.1^*^	6.8 ± 2.7	4.4 ± 2.9
PLT [10^3^/µL]	265 ± 241.9^*^	232.1 ± 75.5^*^	211.3 ± 35.6
LDH [U/L]	580 ± 190	520 ± 178	340 ± 66

BVAS, Birmingham Vasculitis Activity Score; VDI, Vasculitis Damage Index; *P < 0.05 in comparison with controls; GC, glucocorticosteroids; CYC, cyclophosphamide; GPA, granulomatosis with polyangiitis; PBMC, peripheral blood mononuclear cells; CRP, C-reactive protein; PMN, polymorphonuclear cells; PLT, platelet; Ig, immunoglobulin; SD, standard deviation; membr., membranes. Platelet count presented as median and interquartile range. #Five patients in the active-GPA group were treated with cyclophosphamide (cumulative dose [5–37]); all patients at remission of GPA previously received cyclophosphamide.

### 2.2 Neutrophil isolation

Neutrophils were isolated from anticoagulated blood using a negative magnetic separation kit. (EasySep Human Neutrophil Enrichment Kit, STEMCELL Technologies Inc., Canada). Isolated cells were suspended in Hanks’ balanced salts solution (HBSS) with calcium and magnesium-containing 2% fetal bovine serum (FBS, Sigma-Aldrich Chemical Co., USA) for the spontaneous DNA release assay or suspended in lysis buffer for targeted phosphoproteomics measurement.

### 2.3 Neutrophil DNA-release assay

Quantification of spontaneously released dsDNA by isolated neutrophils was performed in 10x10^5^cells cultured for 3 h in HBSS (250 µL, with calcium and magnesium and 2% FBS) at 37°C, 5% CO_2_. Next, DNase I (2.5 U/ml, EURx, Gdańsk, Poland) was added and the reaction was stopped after 10 minutes by the addition of EDTA (pH 8.0, Sigma-Aldrich Chemical Co, USA) to the final 2.5 mM concentration. After sedimentation of cells (190 g, 5 min, 4°C), the concentration of oligonucleotides in the supernatant was measured by fluorometry (Quant-iT Pico Green dsDNA Assay, Thermo Fisher Scientific, USA) in Qubit 3 fluorimeter and expressed as relative fluorescence units (RFU).

### 2.4 Targeted phosphoproteomics of mTOR-related proteins

Levels of selected phosphoproteins: Akt^(Ser473)^, BAD^(Ser136)^, GSK-3α/β^(Ser21/Ser9)^, IRS-1^(Ser636/Ser639)^, mTOR^(Ser2448)^, p70 S6 kinase^(Thr389)^, PTEN^(Ser380)^, S6 ribosomal protein ^(Ser235/Ser236)^), and ULK1 ^(Ser555)^ were measured using a commercially available Bio-Plex Pro™ Cell Signaling Akt Panel (8-plex, Bio-Rad, USA) in Luminex instrument or ELISA (PathScan^®^ Phospho-ULK1, Cell Signaling Technology, USA). Briefly, the total cellular protein was isolated from neutrophils (5x10^6^ cells) with the lysis buffer according to the manufacturer’s protocol, and 10 µg of the protein lysate per well was used for the Luminex assay or ELISA.

### 2.5 Isolation and measurements of circulating cell-free DNA in blood

Total circulating cell-free DNA was isolated from serum using the phenol/chloroform method (8). In brief, 500 µL of serum was spiked with 0.34 ng of an internal standard of DNA (plasmid pGEM-3Zf(+), Promega, Madison, USA) to compensate for errors in sample processing, diluted with 1 mL of ultrapure water, and extracted four times with the same volume of phenol/chloroform mixture. DNA was recovered by precipitation with 1:10 v/v 3M sodium acetate and 2:1 v/v cold 96% ethanol. After rinsing with 70% ethanol and drying DNA, the pellet was resuspended in 20 µL of water. Circulating cell-free mitochondrial (mtDNA) and nuclear DNA (nDNA) were measured separately by quantitative PCR (qPCR, 7900HT real-time PCR system, Applied Biosystems, Foster City, USA). Details of qPCR reactions and primer sequences for genomic DNA (*Alu* repeats target) and mitochondrial 16s RNA gene (mtDNA79) were as previously described (5). Quantification of the internal standard was performed using M13 primers (Promega). The quantification cycles of mtDNA and nDNA were corrected for the internal standard and calculated as relative expression (RE) with the use of the 2^-ΔCt^ formula.

### 2.6 Quantification of circulating DNA-MPO complexes

Circulating complexes of neutrophil myeloperoxidase with DNA were measured using ELISA as previously described (9). Briefly, 96-well ELISA plates were coated with anti-MPO antibody (5µg/mL, AbD Serotec, USA), and a Death plus EIA kit (DNA-Death plus, Roche Switzerland) was used as a source of all buffers and of a secondary, HRP-conjugated antibody. Raw results were presented as mean optical densities (OD).

### 2.7 Screening for differentially expressed circulating proteins in patients with GPA and controls

Profiles of circulating cytokines were compared between active GPA and HC using Proteome Profiler Human XL Cytokine Array Kit (Bio-Techne, Minneapolis, USA). Aliquots of 100 µL of serum from five participants randomly selected from each group were pooled, and then diluted to the final volume of 1.5 mL and used for overnight incubation of membranes spotted with capture antibodies. After washing, the membranes were incubated with detection antibodies and developed with a chemiluminescent reagent. Images were collected using C-DiGit Blot Scanner (Li-Cor Bioscience, Lincoln USA). Optical densities of the detection spots (**Supplementary Figure 1**) were used to select proteins subsequently measured in the serum of all study participants by a custom Luminex multiplex assay (Bio-Techne). Quantified plasma levels of IGFBP-2, IGFBP-3, TFF-3, DPP-IV, basigin (CD147), and CHI3L1 were interpreted from the calibration curves and presented in pg/mL or ng/mL.

### 2.8 Statistical analysis

Statistical calculus was done using GraphPad Prism 9.0 software (GraphPad Software Inc., San Diego, USA). Descriptive statistics was presented as a mean and standard deviation. Differences between the groups were tested by one-way analysis of variance (ANOVA) with Tukey’s *post hoc* test. The goodness of fit for the canonical discriminant function was evaluated by Wilks’s Lambda. Type I statistical error *P* < 0.05 was considered significant.

## 3 Results

### 3.1 Neutrophils dsDNA release and levels of circulating mtDNA, nDNA, and DNA-MPO complexes

**Figure 1:**
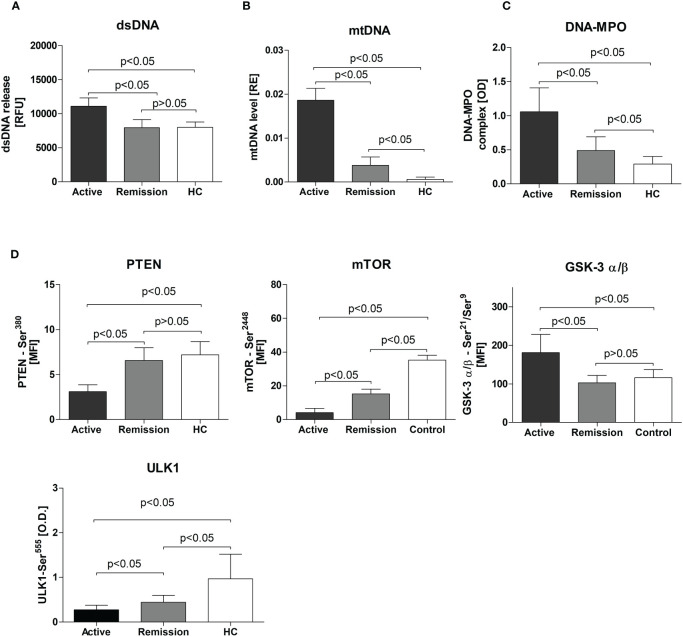
**(A)** Release of dsDNA by resting neutrophils isolated from the study participants. Neutrophils were cultured for 3 hours in HBSS (with calcium and magnesium and 2% fetal bovine serum, at 37°C, and 5% CO2) and the dsDNA content in the supernatant was measured using the fluorimeter. **(B)** Serum level of circulating mitochondrial DNA in study participants - Results are presented as relative expression (RE) calculated using 2^-ΔCt^ formula (ΔCt = Ct_mtDNA_-Ct_pGEM_) from the DNA plasmid spike-in standard (pGEM). **(C)** Circulating DNA-MPO complexes in patients with active GPA, remission, and healthy controls, **(D)** Levels of phosphoproteins: PTEN, mTOR, GSK-3 σ/β, and ULK1 in neutrophils isolated from patients with active stage of GPA, remission of GPA, and healthy controls. All data are presented as the mean and standard deviation (SD).

We observed elevated dsDNA release from non-stimulated neutrophils isolated from patients with active GPA (active: 11070 ± 1233, remission: 7941 ± 1177, HC: 7996 ± 771.7 RFU, p<0.05) (**Figure 1A**). A similar finding was notable for circulating mtDNA (active: 0.01860 ± 0.003, remission: 0.004 ± 0.002 and HC: 0.0006 ± 0.0005 RE, p<0.05) (**Figure 1B**) and DNA-MPO complexes (active: 1.058 ± 0.35, remission: 0.5 ± 0.2, HC: 0.3 ± 0.11 OD, p<0.05) (**Figure 1C**). However, no differences between studied groups were observed in nuclear DNA (nDNA) release (not shown).

### 3.2 Phosphoproteins levels in isolated neutrophils from the study participants

Levels of phosphorylated mTOR^(Ser2448)^, PTEN^(Ser380)^, and ULK1^(Ser555)^ were significantly lower in neutrophils from patients with GPA. This was more apparent for mTOR^(Ser2448)^ levels (active 4 ± 0.5, remission: 15.2 ± 0.6, HC: 35 ± 2.9 MFI, p<0.05) (**Figure 1D**) but also significant for PTEN^(Ser380)^ (active: 3.11 ± 0.7, remission: 6.6 ± 1.4, HC: 7.2 ± 1.5 MFI, p<0.05) (**Figure 1D**) and ULK1^(Ser555)^(active: 0.28 ± 0.1, remission:0.44 ± 0.15, HC: 0.99 ± 0.55, p<0.05) (**Figure 1D**). In patients with active disease, a significant elevation of phosphorylated GSK-3α/β^(Ser21/Ser9)^ was detected (active: 181 ± 47.4, remission: 102.5 ± 19.12, HC: 116.1 ± 20.83 MFI, p<0.05) (**Figure 1D**). No differences between the study groups were observed in five phosphoproteins measured: Akt^(Ser473)^, BAD^(Ser136)^, IRS-1^(Ser636/Ser639)^, p70 S6 kinase^(Thr389)^, and S6 ribosomal protein^(Ser235/Ser236)^) (not shown).

### 3.3 Levels of differentially expressed serum proteins in GPA

**Figure 2:**
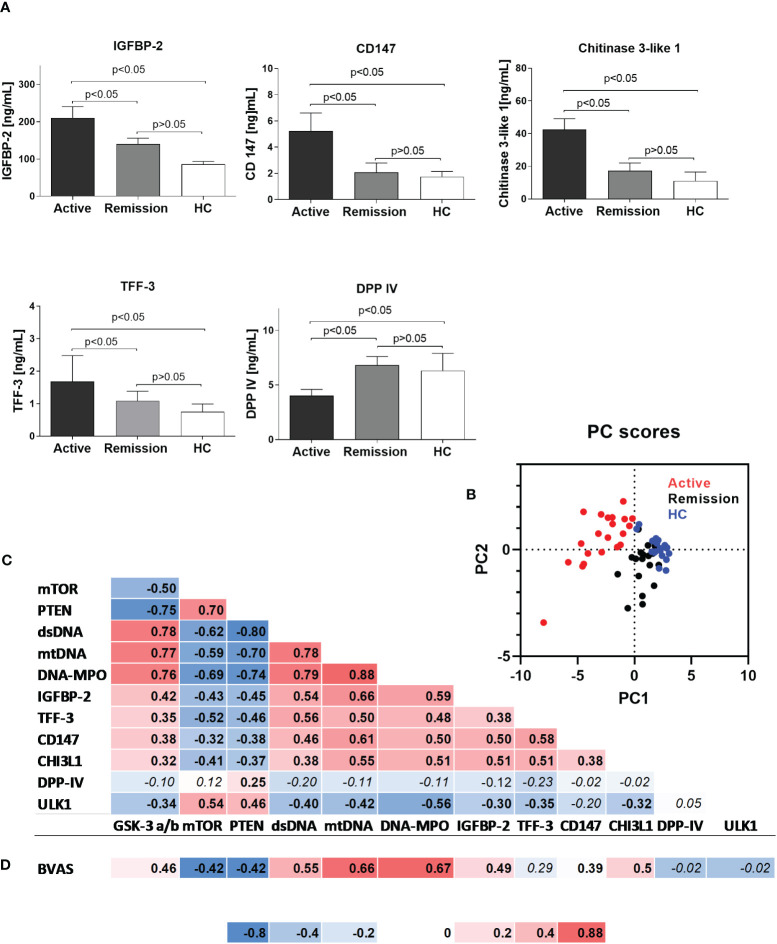
**(A)** Serum levels of selected proteins in the study participants. Results are presented as the mean and standard deviation (SD). **(B)** Canonical discriminant function plot of analyzed parameters in patients with active GPA (n=21, red), remission of GPA (n=21, black), and healthy control (n=21, blue). Wilks’s Lambda V=0.067, p<0.0015. **(C)** Correlations between analyzed parameters in all study participants (correlation was evaluated using Pearson correlation, p<0.05). **(D)** Correlation between BVAS score and analyzed parameters in the group of patients with active stage of granulomatosis with polyangiitis (correlation was evaluated using Pearson correlation, p<0.05). Non-significant *P* values >0.05 are marked in italics.

Using a semiquantitative protein array, we performed a screening for differentially expressed serum proteins in patients with active GPA vs. healthy controls. The results suggested altered circulating levels of IGFBP-2, IGFBP-3, TFF-3, DPP-IV, CD147, and CHI3L1 (**Supplementary Figure 1**). Measurements of the selected proteins in all the participants of the study revealed that in the active stage of GPA, levels of IGFBP-2 were the highest (active: 210 ± 30, remission: 140 ± 15 and HC: 85 ± 8 ng/mL, p<0.05) (**Figure 2A**). Significant elevations also characterized TFF-3 (active: 1.6 ± 0.7, remission: 1.9 ± 0.3 and HC: 0.7 ± 0.2 ng/mL, p<0.05), CD147 (active: 5.2 ± 1.3, remission: 2.0 ± 0.7 and HC: 1.7 ± 0.4 ng/mL, p<0.05), and CHI3L1 (active: 42.5 ± 6, remission: 17 ± 4.5 and HC: 11 ± 5 ng/mL, p<0.05) (**Figure 2A**). However, DPP-IV levels were the lowest in the serum of patients with the active stage of GPA (active: 4 ± 0.5, remission: 6.8 ± 0.7 ng/mL and HC: 6.3 ± 1.5 ng/mL, p<0.05).

### 3.4 Correlations of analyzed phosphoproteins and serum proteins with biomarkers of NET formation

Principal component analysis of differentially expressed phosphoproteins and serum proteins allowed for an efficient (98% correct) classification of all participants of the study into active or remission GPA or HC. Only a single participant from the healthy control group was incorrectly classified as GPA at remission (Wilks’s Lambda V = 0.067, P < 0.001, **Figure 2B**). A discrimination matrix was tested using a jackknife subsampling and was correct for 92% of participants. PCA vectors explained 66% of the variance and had canonical correlations of 0.92 and 0.75. Positive correlations were observed between biomarkers of NETosis (circulating mtDNA, DNA-MPO complexes levels, and dsDNA level) and the circulating proteins (IGFBP-2, TFF-3, CD147, and CHI3L1) or phosphorylated GSK-3 α/β. Phosphorylated mTOR and PTEN correlated negatively (**Figure 2C**) with NETosis biomarkers or circulating proteins. A very similar pattern of correlation matrix characterized BVAS, which correlated positively with dsDNA, mtDNA, DNA-MPO complexes, IGFBP-2, CD147, CHI3L1, and phosphorylated GSK-3 α/β, and negatively with phosphorylated mTOR and PTEN (**Figure 2D**).

## 4 Discussion

This study focused on the levels of mTOR phosphoproteins signaling pathway involved in neutrophil NETosis. Itakura et al. postulated a pivotal role of mTOR-regulated autophagy in the control of NETosis (10). Autophagy was activated by inhibition of mTOR and accompanied by increased NETosis. Copp et al. (11) found mTOR activity controlled by phosphorylation status rather than variable intracellular protein level. Protein kinase B (PKB) phosphorylates mTOR at serine 2448 (Ser^2448^), activating this pathway. In our study, a lower level of mTOR phosphorylated at Ser^2448^ was detected in neutrophils isolated from patients with active GPA. Moreover, mTOR suppression negatively correlated with BVAS, measuring the disease activity. Elevated biomarkers of GPA activity, such as circulating mtDNA, DNA-MPO complexes, or resting dsDNA released by neutrophils, were in line with this finding. Lowered mTOR^(Ser2248)^ was accompanied by high levels of phosphorylated glycogen synthase kinase-3 - GSK-3 α/β. This enzyme is active in resting cells, suppressing the signaling of multiple pathways, including Wnt/β-catenin, JUN, and glycogen synthase. GSK-3 α/β activity is inhibited by its phosphorylation at serine 21 and 9 (Ser^21/9^). Giambelluca et al. reported in neutrophils that stimulation of the cells with an agonist inducing NETosis led to phosphorylation of GSK-3α ^Ser21 (^ 12). In agreement with this report, we documented higher phospho-GSK-3 levels in both subunits of the kinase complex. At physiological conditions, GSK-3 can indirectly inhibit the mTOR pathway by phosphorylation of a common substrate tuberin (TSC2). Therefore, the positive correlation of phospho-GSK-3 with BVAS or NETosis biomarkers suggests a profound alteration of intracellular signaling of neutrophils in GPA. Whether this mechanism relies on a lack of response to metabolic or hormonal cues or activated autophagy requires further studies. By the targeted phosphoproteomics approach, we also found decreased levels of phosphorylated phosphatase and tensin homolog deleted on chromosome 10 (PTEN) and Unc-51-like kinase 1 (ULK1). Phosphorylation of PTEN at serine 380 (Ser^380^) increases the stability of the protein but inhibits its phosphatase activity. PTEN can increase NETosis in a mechanism linked to the autophagy process (13). Lower PTEN^Ser(380)^ found in GPA negatively correlated with markers of NETosis. ULK1 is a well-known autophagy regulator that can be controlled by AMP-activated protein kinase (AMPK) and mTOR. Several studies described AMPK activation-mediated ULK1 phosphorylation as an autophagy-stimulating process (14, 15). However, AMPK activation can also lead to autophagy inhibition (16). Moreover, low activity of mTOR can be linked to low activity of ULK1 (17). In the case of our study, in neutrophils from patients with GPA, we reported lower phosphorylation of mTOR and lower phosphorylation of ULK1, which seems to confirm previous observations. Interestingly, we could not distinguish which intracellular mTOR complex was inhibited in neutrophils. Ribosomal proteinS6 kinase- β_1_ phosphorylated at threonine 389 (p70 S6 kinase^Thr389^), a downstream signal of mTORC1, and protein kinase B member Akt1 phosphorylated at serine^473^ (Akt^Ser473^) downstream to mTORC2 did not differ between the study groups. A further link to increased autophagy in neutrophils from GPA patients resulted from differentially expressed serum proteins. Four out of five proteins selected during a preliminary screening, namely, insulin-like growth factor binding protein 2 (IGFBP-2), basigin (CD147), chitinase-3-like protein 1 (CHI3L1), and trefoil factor 3 (TFF-3), were elevated in the serum of patients with active GPA, whereas dipeptidyl peptidase-IV (DPP-IV) was significantly decreased. IGFBP-2 is a member of insulin-like growth factor binding proteins (IGFBPs) accompanying insulin-like growth factors. Increased serum IGFBP-2 levels were detected in patients with systemic lupus erythematosus, rheumatoid arthritis, or inflammatory bowel disease (18). Its role in neutrophil activation and NETosis is unclear but can be related to enhanced autophagy (19). It is a limitation of our study that we did not analyze autophagy in neutrophils, but serum levels of IGFBP-2 correlated with biomarkers of NETosis and phosphoproteins of mTOR, GSK-3α/β, PTEN, and ULK1. The other three proteins elevated in the serum of patients with GPA can regulate autophagy. TFF-3 and TFF-1 are members of the trefoil factor (TFF). Down-regulation of TFF-3 prevented autophagy in colon cancer adenocarcinoma (20). Basigin (CD147) is a transmembrane glycoprotein expressed in numerous cell types. It is mandatory for intracellular recognition and the proper placement of membrane proteins. Increased surface expression of CD147 was observed in neutrophils from patients with rheumatoid arthritis (21). In prostate cancer, CD147 regulates autophagy via the PI3K/Akt/mTOR pathway (22). CHI3L1 belongs to the glycoside hydrolase family-18 and is secreted by various cells, including activated neutrophils. Recently a high level of circulating CHI3L1 was reported in GPA (23). In the present study, the highest level of CHI3L1 was detected in patients with GPA exacerbation and correlated with BVAS. DPP-IV is a serine exopeptidase modulating immune and metabolic responses. Interestingly, inhibition of this enzyme by gliptins is not only beneficial in diabetes but is also considered to have anti-inflammatory effects. Therefore, this finding requires further studies. In conclusion, the results of this study evidenced inhibition of the mTOR signaling pathway accompanied by enhanced NETosis in patients with GPA. It is an important finding for clinicians because mTOR inhibitors are being tested for autoimmune diseases. The elevated level of circulating proteins that we described provides a suggestive link between clinical activity of the disease or NETosis biomarkers and autophagy, which warrants further studies on GPA. As reported by us, differential expression of phosphoproteins and circulating proteins has also some diagnostic potential, as shown by PCA. Unfortunately, we could not present any direct evidence of autophagy involvement in NETosis in GPA. Another limitation of this study is the descriptive interpretation of findings based on the correlation of clinical and laboratory parameters. However, to the best of our knowledge, this is the first report describing targeted phosphoproteomics of the mTOR pathway in neutrophils from patients with GPA.

## Data availability statement

The original contributions presented in the study are included in the article/**Supplementary Material**. Further inquiries can be directed to the corresponding author.

## Ethics statement

The studies involving humans were approved by The Ethics Committee of the Jagiellonian University. The studies were conducted in accordance with the local legislation and institutional requirements. The participants provided their written informed consent to participate in this study.

## Author contributions

MSu - conceptualization, methodology, data curation and analyses, investigation, writing original draft, and review & editing. JK-W, KW-A, and AW – clinical data curation and analyses. MSa – data analyses, supervision, and writing - review & editing. JM - writing - review & editing. All authors contributed to the article and approved the submitted version.
