# Supplementary material for: Activity of granulomatosis with polyangiitis and its correlation with mTOR phosphoproteomics in neutrophils
Source: Front Immunol. 2023 Aug 31;14:1227369. doi: 10.3389/fimmu.2023.1227369 (PMC10500300; doi:10.3389/fimmu.2023.1227369)
Supplement: Supplementary file 1 [file DataSheet_1.docx]

sFIG. 1. Screening of differentially expressed proteins in serum of healthy controls (panel A) and GPA patients with active disease (panel B). Presented results were obtained using Proteome Profiler Human XL Cytokine Array Kit (Bio-Techne, USA).
